# Lipoedema as a Social Problem. A Scoping Review

**DOI:** 10.3390/ijerph181910223

**Published:** 2021-09-28

**Authors:** Monika Czerwińska, Paulina Ostrowska, Rita Hansdorfer-Korzon

**Affiliations:** Department of Physiotherapy, Medical University of Gdańsk, 80-211 Gdańsk, Poland; paulina_graf@gumed.edu.pl (P.O.); rita.hansdorfer-korzon@gumed.edu.pl (R.H.-K.)

**Keywords:** lipoedema, lipedema, obesity, quality of life

## Abstract

(1) Background: Lipoedema is a disease characterized by excessive bilateral and symmetrical accumulation of subcutaneous tissue in the lower extremities. It is a poorly understood condition, and low awareness of its existence often leads to incorrect diagnosis Initially, lipoedema was considered to be completely independent of lifestyle Currently, however, more and more cases of the coexistence of lipoedema and obesity are described in the literature as additionally affecting the severity of the disease The aim of the review is to present lipoedema as a social problem. (2) Methods: Materials on lipoedema in the social context were selected from 2018–2021. The PRISMA-Scr checklist was used in the review. (3) Results: Research has shown that more than 3/4 of patients with lipoedema are also overweight or obese. Patients with lipoedema have many comorbidities, and their presence negatively affects the quality of life. The quality of life in patients with lipoedema is lower than in healthy patients. (4) Conclusions: The number of studies available on lipoedema is low. Obesity is common in patients with lipoedema. Mental disorders increase the level of experienced pain. Lipoedema significantly reduces quality of life. A healthy lifestyle in patients with lipoedema could be helpful for prevention of complications and disability.

## 1. Introduction

As a disease, lipoedema is still not fully understood [1,2,3,4]. So far, the, pathomechanism and the degree of its general prevalence have not been established [4,5,6]. Currently, available data suggest that genetic factors play a very important role in the development of lipoedema [7,8]. Many researchers have attempted to determine the aetiology and mechanism of the development of lipoedema, but the presented data still remain mere hypotheses [1,2,3,4,9,10,11]. The prevalence of lipoedema in the general public is also unknown, and the data from many sources in this regard is widely divergent [9,12,13]. There is also a lack of systematic knowledge of the diagnostic criteria, which often leads to incorrect diagnosis [14]. Difficulties in diagnosis result in a delay or absence of targeted treatment, which results in a much more severe course of the disease leading to disability [1,4,15,16]. Lipoedema is defined as the pathological multiplication of adipose tissue cells due to hormonal change [17]. The disease affects mainly women, and usually begins during puberty, pregnancy or menopause [9]. Isolated cases of lipoedema in men have been described in the literature and hormonal disturbances were present in all cases [9]. Lipoedema is characterized by a bilateral and symmetrical distribution of adipose tissue, mainly around the lower extremities [16,17]. Its specific feature is the presence of a visible disproportion between a slim upper body and thickened lower limbs [11,18]. What is also very important is that attempts to lose weight through intense physical activity and restrictive diets fail to reduce lipoedema, resulting only in the increase in the existing disproportion between the upper and lower half of the body [2,16]. Other features that significantly distinguish lipoedema from other conditions are increased tendency to bruising, heaviness in the legs and high tactile sensitivity of the tissue affected by lipoedema [11,18,19]. Therefore, lipoedema is not only an aesthetic problem but also has a significant impact on the functioning and mobility of the patient [1,4]. Currently, the complexity and multifactorial nature of this disease are more and more emphasized. A major obstacle in the correct diagnosis of this disease is its coexistence with obesity. Excessive body weight in this case may mask some characteristic features of lipoedema. Obesity or being overweight is believed to accompany a very large group of patients with lipoedema, and it significantly worsens the symptoms of lipoedema and the patient’s general condition [1,9,20].

Another important issue is the influence of mental state, both on the development of the initial symptoms of the disease and its role in the severity of the experienced ailments. Patients with lipoedema often face a lack of understanding from their environment, which negatively affects their self-esteem, thus resulting in physical limitation of daily activities [15,16,21]. Lipoedema, due to its chronic and progressive nature, has a significant impact on the quality of life of patients affected by it [22]. Very low awareness of lipoedema among physicians and the public often leads to misdiagnosis. In many cases, proper diagnosis is formulated many years after the symptoms of lipoedema appear [23,24]. Alongside long-term lipoedema lasting a number of years, a mixed form of lipo-lymphoedema may develop. It is an advanced stage of the disease where, in addition to the symptoms typical of lipoedema, the patient successively develops lymphatic insufficiency, lymphoedema and other complications such as erysipelas or lymph ulcer [25]. The aim of this review is to present the current knowledge about lipoedema in the context of it being a social problem by trying to answer the following questions: (1) Is lipoedema related to lifestyle? (2) Does the presence of lipoedema increase the risk of other diseases? (3) To what extent does lipoedema affect quality of life? (4) Does mental state increase the symptoms of lipoedema? 

## 2. Materials and Methods

**Figure 1:**
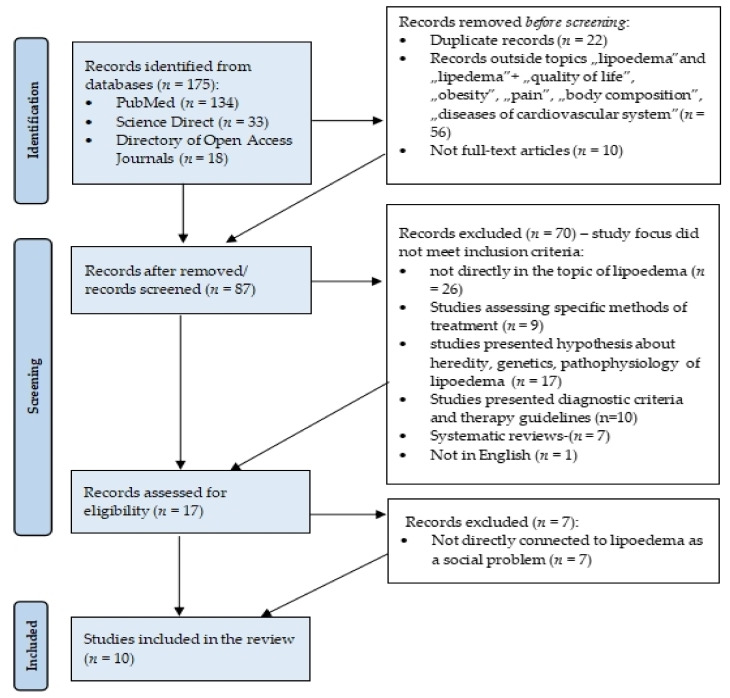
Flow diagram adapted from PRISMA which shows the process for identifying and screening the articles for inclusion and exclusion.

In order to try to answer the above questions, on June 18, 2021, a review of the available literature was carried out with the help of the multi-search engine at the Medical University of Gdansk main library. The review took into account articles published in 2018–2021 in English and available in the following databases: Medline Complete (PubMed), Directory of Open Access Journals, and Science Direct. After searching for “lipoedema or lipedema”, 175 results were obtained (PubMed: 134, Science Direct: 23, Directory of Open Access Journals: 18). Then, the results were narrowed down to the terms “lipoedema “, “lipedema”, “quality of life”, “chronic disease”, “obesity”, “pain”, “body composition”, and “disease of cardiovascular system”, and 119 results were obtained. Subsequently, publications not available in full (*n* = 10) and duplicates (*n* = 22) were removed. The authors of the publication analysed the titles of 87 works. A total number of 70 results was rejected: 26 not directly related to lipoedema; 9 providing information on specific treatment methods (e.g., liposuction); 17 addressing the issues of pathophysiology, aetiology and genetic factors influencing lipoedema; 10 presenting diagnostic criteria and methods; 7 systematic reviews; and one not available in English. Then, 17 full texts of publications were analysed. A total of 10 articles dealing with the issue of lipoedema in the context of it being a social problem were selected. The selection was made on the basis of the following inclusion criteria: 1. studies in which patients with diagnosed lipoedema participated; 2. publications presenting quantitative clinical studies, case reports, clinical observations or qualitative studies; 3. publications containing data on lipoedema in the social context with reference to quality of life, comorbidity, connection with body weight, and connection with mental state; 4. publications in English. Data were extracted from patients’ BMI, comorbidities, quality of life, psychological distress connected with lipoedema, and time between onset and diagnosis of lipoedema. The question generated with the use of PICOS components (Population, Intervention, Comparison, Outcomes, Study Design) was: “In patients with lipoedema (P), a healthy (active) lifestyle combined with an appropriate diet and pharmacotherapy (I) is an effective physiotherapeutic tool that influences the improvement of quality of life (O) in relation to patients in the control group (C)?”. Articles using questionnaires assessing the quality of life of patients with lipoedema (S) were included in the qualitative synthesis. The research was conducted using PRISMA methodotogy (Figure 1)

The review was conducted according to the PRISMA method and it distinguished 10 papers, which are presented in Table 1.

## 3. Results

### 3.1. Lipoedema and Body Weight Relationship

Body weight is assessed using the BMI, which is the ratio of body weight in kilograms to height, expressed in meters and squared. According to WHO, obesity is found at values above 30 kg/m^2^, overweight 25–29.9 kg/m^2^ and normal body weight 18.5–24.9 kg/m^2^. 

Four studies reported on the BMI of patients, and in all studies the largest proportion were classified as obese [20,23,26,27]. This was greatest in the study by Erbacher (*n* = 150, 76.7%) who also reported the smallest proportion of participants with BMI in the normal range (3.3%) [23]. The most recent study by Dudek reported the largest proportion of patients in the obese category (*n* = 95, 50%) and the least proportion of normal body weight cases (20.4%) [26]. The BMI of the patient in the earlier study by Dudek and the study by Romejn fall between these ranges [23,27]. All BMI values are summarised in Table 2.

Thomas F. Wright and Karen L. Herbst present a case report of a young woman with lipoedema. The first symptoms in the form of accumulation of adipose tissue around the thighs, calves and ankles appeared in her at the age of 12, and despite normal BMI (17 kg/m^2^), the disturbance in proportion was clearly marked. The woman, thinking she was obese, tried to lose weight using very restrictive diets and intense physical activity. Despite a significant reduction in body weight (BMI 15 kg/m^2^), the disproportion was still visible, and the woman developed anorexia. When she regained normal body weight (BMI 21 kg/m^2^), other symptoms appeared in the form of pressure sensitivity and tendency to bruising [29].

### 3.2. Lipoedema Associated Comorbidities

In 2019, a study was conducted in Korea to investigate the relationship between obesity and the development of subclinical and clinical lymphoedema in patients with lipoedema. A total of 258 women with lipoedema participated in the study. The subjects were divided into 3 groups according to BMI. Group 1 had 98 women with BMI less than 30 kg/m^2^, group 2 had 124 with BMI between 30 and 40 kg/m^2^, and group 3 had 36 women with BMI over 40. The occurrence of lymphoedema was assessed using bioimpedance. Subclinical lymphoedema was found least in subjects from group 1 (16.3%) and increased with increasing BMI to 48.3% in group 2 and 72.3% in group 3. Likewise, clinical lymphoedema was found least in subjects from group 1 (6.1%) and increased with increasing BMI to 51.6% in group 2 and to 77.8% in group 3. This proves a significant relationship between the occurrence of obesity and the appearance of lymphoedema in patients with lipoedema [25].

In our review, we found no studies on comorbidities associated with lipoedema. The only data on the subject originates from questionnaire studies in which patients with lipoedema voluntarily report their diseases.

Four studies reported on the presence of comorbidities and all indicated the presence of hypothyroidism among their cohorts [23,24,26,30]. Overall, the largest proportion of patients included in these studies were obese (28%), and 27.5%, 22.7%, and 19.2% were affected by hypothyroidism, depression, and allergies respectively. Sleep disorders and migraines were present in 12.5% of subjects followed by hypertension (10%) and asthma (9.4%). Bowel disorders were reported by 8.7% of patients, and 6.25% of patients suffered from rheumatic diseases. Out of all respondents, 6.2%, 6%, and 5.56% were affected by lymphoedema, heart disease, and diabetes respectively. The least portion of subjects reported dyslipidemia (3.8%), polycystic ovary syndrome (3.5%), skin disorders (3%) and fibromyalgia (3.2%). One quarter of all subjects had no comorbidities (25.2%). Table 3 presents the number of comorbidities in people with lipoedema.

Nemes et al. conducted a study to assess whether lipoedema was associated with the occurrence of disorders in the structure of the left ventricle. The study included 19 women with lipoedema and 28 healthy women (control group) matched to the study group in terms of age. To evaluate the heart muscle, echocardiography was performed. Compared to the control group, in patients with lipoedema, dimensions of the left atrium and the left ventricle were larger, likewise the ejection fraction was larger. The diameter of the left atrium in the control group was 34.4 ± 4.1 mm, and in the patients with lipoedema it was 39.6 ± 4.1 mm. The diameter of the left ventricle in the control group was 45.8 ± 3.2 mm, and in the study group it was 49.8 ± 3.2 mm. The ejection fraction in the control group was 64.3 ± 4.3%, and in the study group it was 68.3 ± 3.6% [31].

### 3.3. Quality of Life

Three articles were found presenting quality of life surveys of patients with lipoedema. In order to assess the quality of life of the respondents, two publications used the WHOQOL-BREF (The World Health Organization Quality of Life) form, one used RAND 36, and one used EQ5 DL3 L [23,26,27].

J R.M Romejn et al. conducted a study in the Netherlands to check the quality-of-life level among patients with lipoedema using the EQ-5 D-3 L and RAND-36 survey forms. EQ-5 D-3 L analyses quality of life from the viewpoint of health. The questionnaire consists of five parts, and assesses mobility, independence, daily activities, pain, and depression levels. The mean value of the EQ-5 D-3 L index was lower (66.1/100) in patients with lipoedema compared to the general Dutch population (85/100). Daily activities such as work, study, and household chores were difficult for 64.8% of the respondents, while 3.1% were completely unable to perform these tasks. Disorders of the musculoskeletal system occurred in 63% of the respondents, but only 9.9% reported difficulties with washing and dressing, and 1.2% of the respondents were unable to get dressed without assistance. The occurrence of ailments involving pain was confirmed by 74.1% of patients, and 16.7% described the pain as severe. Depression was found in 42% of respondents [23].

The same study also used another form to assess quality of life, namely RAND-36. It is a 36-item questionnaire assessing nine aspects of health: perception of general health; physical functioning; mental health; pain; impact of limitations stemming from emotional problems; and dysfunctions caused by physical limitations, social functioning, vitality, and changes in health status. The total RAND score differed significantly between patients with lipoedema (59.3/100) and the mean score in the Dutch population (74.9/100) [23].

A study published by Dudek in 2018, which included 329 patients with lipoedema, showed a relationship between the quality of life and a more severe course of the disease, depression, and reduced mobility. An inverse relationship was found between quality of life and severity of symptoms of lipoedema (r = −0.651), severity of depression (r = −0.75), and stress level stemming from physical appearance (r = −0.654). It was also observed that the higher the mobility, the better the perception of quality of life (r = −0.607), where r is the relationship between two variables, i.e., the strength of the correlation [27].

Dudek et al. conducted another study with the participation of 98 women with lipoedema. The WHOQOL-BREF form was used to assess the quality of life in four aspects: physical health, mental health, social relations, and the environment. The maximum number of points in each part was 100. The mean value of quality of life with respect to physical health was 45.4; with respect to mental health, it was 46.3; with social relations, 50.4; and with the environment, 49.6 [26]. Table 4 presents the summary and characteristics of the above-mentioned studies.

Melander C. et al. conducted a qualitative study which involved interviewing 14 women with lipoedema. The main goal was to collect information on the experiences of women diagnosed with lipoedema, and the influence of this disease on their subjective view on health. The study participants stated that lingering pain significantly affected their daily functioning. The respondents admitted that each step meant an enormous effort, and constant feelings of heaviness and pain in the lower limbs brought a feeling of powerlessness and lack of control over the body. Moreover, the respondents emphasized lack of understanding on the part of physicians who downplayed the symptoms and recommended vigorous physical activity, diet, and diuretics [28].

### 3.4. Time of Diagnosis

The authors of the review found two articles presenting data on the time between the onset of lipoedema symptoms and its correct diagnosis [23,24].

Both studies found that symptoms first appeared on average during adolescence and young adulthood (range 16–20 years), while diagnosis occurred many years later (range 15–18.3 years) [23,24].

Table 5 presents average waiting time for diagnosis of lipoedema.

### 3.5. Psychological Stress and Pain

Erbacher and Bertsch conducted a study to check the impact of psychological stress on the development of symptoms of lipoedema. Two interviews were conducted with each of the 150 respondents. For the purposes of the study, psychological stress was defined as the presence of a mental illness (according to ICD-10:eating disorders, depression, anxiety disorders, post-traumatic stress disorder, and panic attacks) or the presence of symptoms that may indicate a mental illness. The interview also included the severity of lipoedema symptoms and the severity of pain. Depressive disorders, eating disorders, post-traumatic stress disorder, and anxiety disorders occurred in 51,1% of the respondents. In addition, it was also noted that the above diseases were more common in patients with BMI higher than 40 kg/m^2^ compared to those with a lower BMI. Pain intensity was measured using a 10-point VAS (Visual Analogue Scale), where 0 is no pain and 10 is maximum pain. No pain and very slight pain (1 point on the VAS scale VAS) were not indicated by any of the respondents. The value of 2 points on the VAS scale was indicated by 0.7% of the respondents, 3 by 2.7%, 4 by 13.3%, 5 by 14%, 6 by 18%, 7 by 17.3%, and 8 by 19.4%; pain intensity at 9 points on the VAS scale was indicated by 8.7% of the respondents, and the maximum level of perceived pain occurred in 6% of the respondents. After comparing the level of pain with the occurrence of psychological stress, it was shown that in patients with mental disorders, the level of perceived pain on the VAS scale was higher (on average, 6.95 points on the VAS scale in patients with mental disorders, 6.32 in patients with no mental disorders) [20].

## 4. Discussion

According to the currently available data, it cannot be unequivocally stated that lipoedema is responsible for weight gain or that obesity and being overweight contribute to the development of lipoedema [1,4]. 

The authors’ analysis shows that body weight in approximately 74% of patients with lipoedema indicates obesity. Observations conducted by Bertsch in a German clinic show that 88% of 2344 patients are obese [1]. Additionally, a study published by K. Herbst in 2015 showed that 76.1% of 46 subjects with lipoedema were obese [32]. Another study conducted by A.H. Child et al. showed that out of 67 patients with lipoedema, only 4% presented normal body weight, 11% were overweight and as many as 85% of subjects had obesity [9].

One of the characteristic features of lipoedema is the apparent disproportion between the trunk and the lower limbs [1,4,29,33,34]. It is precisely this initially slight disproportion that is often the first symptom of lipoedema, appearing in adolescence. At this stage, women do not report any soft tissue ailments such as pain and bruising [1].

With time and with the gradual increase in body weight, the risk of developing further symptoms of lipoedema increases [5] Women with lipoedema often undertake initial, unskilful efforts to lose weight in adolescence, and this leads to a spiral of attempts to lose weight lasting for many years, resulting in more and more weight gain [5].

It should be emphasized that there is a group of patients who present symptoms of lipoedema despite having a normal body weight. In these patients, attempts to lose weight are unsuccessful because the subcutaneous tissue accumulated on the lower limbs is resistant to slimming, and losing weight only increases the disproportion between the torso and the limbs [29]. Moreover, due to difficulties in losing weight for many women with lipoedema, eating disorders in the form of anorexia and bulimia develop [29]. Survey research carried out by Lipoedema UK showed that out of 250 women with lipoedema, 45% of respondents report having eating disorders such as over- or under-eating, anorexia, bulimia, and binge eating [35].

Obesity significantly increases the risk of lymphoedema, and thus, other related complications [25]. Recurrent bacterial and fungal infections and recurrent cellulitis and lymphangitis are complications of lymphoedema, leading to increasing damage of the lymphatic system and lymph ulcers [36]. The coexistence of lymphoedema and lipoedema causes great discomfort and pain, and frequent infections significantly reduce the patients’ quality of life [25].

So far, no detailed studies on the relationship between lipoedema and the occurrence of other diseases have been carried out. Of the studies presented in this review, 25.17% of subjects with lipoedema reported no other diseases. The most frequently indicated diseases were obesity (28%), hypothyroidism (27.5%), and depression (22.7%). There was a relatively large difference in the prevalence of hypothyroidism in patients with lipoedema (27.5%) compared to the prevalence of this disease among the general population (0.5–2%). The relationship between the above diseases is not fully explained, but it can be assumed that hormonal disorders have a significant influence on lipoedema [30]. 

Lipoedema significantly affects the quality of life [22,23,26,27,37]. The studies presented by the authors show a relationship between the severity of symptoms and a lower level of quality of life. In a 2014 Lipoedema UK study, out of 242 patients with lipoedema, 211 (87%) of respondents admitted that lipoedema had a negative impact on quality of life, 28 (12%) indicated that lipoedema did not affect quality of life, and 3 (1%) said it had a positive effect on quality of life. In addition, 86% of respondents admitted that lipoedema caused a decrease in self-esteem, 60% reported a reduction in social activities, 60% indicated a feeling of helplessness, 47% a feeling of loneliness, and 55% significant limitation of the capability of the musculoskeletal system [35].

In the studies selected by the authors, a relationship was observed between mental disorders and the level of perceived pain in patients with lipoedema. Stigmatization, labelling, and downplaying of suffering affects lipoedema patients’ mental states, which makes it difficult to implement effective therapy and increases the level of experienced pain [20,38].

The importance of the influence of genetic factors on the occurrence of lipoedema should also be emphasized. The latest research carried out by S. Michelini et al. indicates a specific gene that may be closely related to lipoedema (AKR1 C1). It is the gene that encodes the aldo-keto reductase that catalyzes the reduction of progesterone to an inactive form. The analysis carried out by the researchers suggests that a mutation in this gene results in a partial loss of aldo-keto reductase function. This results in a slower and less efficient reduction of progesterone to 20-α-hydroxyprogesterone, and this could result in increased deposition of subcutaneous fat. Taking into account the latest reports, the possibility of targeted pharmacological treatment in people with lipoedema should be noted. Understanding the genes underlying lipoedema would allow the introduction of pharmacological treatment that would directly affect related or causative molecule [8].

The main limitation of this study was lack of randomised controlled trials dealing with the topic of lipoedema as a social issue. Most of the studies included in this review used surveys filled out by patients, which were not absolutely objective. Further studies should be carried out to establish the connection between lipoedema, lifestyle, comorbidities, and quality of life. 

## 5. Conclusions

A majority of lipoedema patients is also overweight or obese and with the increase in BMI, the risk of lymphoedema in patients with lipoedema also increases. Lipoedema significantly reduces the level of quality of life, and there is a relationship between the level of perceived pain and the occurrence of psychological distress in lipoedema patients.

Further research should be carried out to increase knowledge about lipoedema and to establish specific diagnostic criteria, which would decrease the waiting time for diagnosis. There is a need to carry out more clinical trials in which patients with diagnosed lipoedema participate. Currently, the number of randomized controlled trials concerning lipoedema patients is low, and as mentioned before, the survey research and clinical observations are not absolutely objective.

## Figures and Tables

**Table 1:** Description of articles initially included by PRISMA methodology.

Article Type	Focus	Reference
Cross sectional study	● Quality of life of people with lipoedema	[26]
	● Quality of life of people with lipoedema	[27]
	● Quality of life of people with lipoedema	[23]
	● Correlation between BMI and lymphoedema in people with lipoedema	[25]
	● Lipoedema comorbidities	[24]
Qualitative research	● Subjective self-evaluation of the impact of lipoedema on health	[28]
Case study	● Lipoedema and eating disorders	[29]
Pilot study	● The connection between pain and psychological stress in people with lipoedema	[20]
Other study	● Lipoedema comorbidities	[30]
	● Left atrial and ventricular abnormalities in people with lipoedema	[31]

**Table 2:** Body weight in people with lipoedema.

Reference	Number of Subjects	Data not Available	Normal Body Weight (BMI = 18.5–24.9 kg/m^2^)	Overweight (BMI = 25–29.9 kg/m^2^)	Obesity (BMI > 30 kg/m^2^)
[27]	*n* = 329	*n* = 8	14 (4.3%)	40 (14.4%)	267 (81.3%)
[26]	*n* = 98	*n* = 3	20 (20.4%)	26 (26.5%)	49 (50%)
[20]	*n* = 150	*n* = 0	5 (3.3%)	15 (10%)	130 (86.7%)
[23]	*n* = 163	*n* = 0	21 (12.9%)	41 (25.2%)	101 (61.9%)
Total	*n* = 729	*n* = 11	60 (8.23%)	122 (16.73%)	547 (75.04%)

**Table 3:** Occurrence of comorbidities among people with lipoedema.

Comorbidity	Ghods M. [30] (*n* = 106)	Dudek J.E. [26] (*n* = 98)	Bauer A.T. [24] (*n* = 209)	Romejn J.R.M. [23] (*n* = 163)	Total (*n* = 576)
Allergy	39 (36.8%)	-	72 (34.4%)	-	111 (19.27%)
Obesity	39 (36.8%)	49 (50%)	-	81	161 (28%)
Sleep disorders	27 (25.5%)	-	45 (21.5%)	-	72 (12.5%)
Hypothyroidism	33 (31.1%)	31 (31.6%)	75 (35.9%)	19 (11.65%)	158 (27.5%)
Depression	27 (25.5%)	56 (57.14%)	48 (23%)	-	131 (22.74%)
Hypertension	26 (24.5%)	4 (4.1%)	28 (13.4%)	-	58 (10%)
Migraine	24 (22.6%)	-	47 (22.5%)	-	71 (12.5%)
Skin disorders	20 (18.8%)	-	-	-	20 (3.5%)
Asthma	19 (18%)	-	27 (12.9%)	8 (4.9%)	54 (9.4%)
Bowel disorders	11 (10.4%)	4 (4.1%)	27 (12.9%)	8 (4.9%)	50 (8.7%)
Rheumatic diseases	9 (8.4%)	20 (20.4%)	7 (3.3%)	-	36 (6.25%)
Dyslipidemia	7 (6.6%)	-	15 (7%)	-	22 (3.8%)
Diabetes (type 1 and 2)	5 (4.7%)	13 (13.3%)	5	9 (5.5%)	32 (5.56%)
Polycystic Ovary Syndrome	3 (2.8%)	5 (5.1%)	12 (5.7%)	-	20 (3.5%)
Lymphoedema	-	30 (30.6%)	-	5 (3%)	35 (6.2%)
Venous insufficiency	-	20 (20.4%)	-	-	20 (3.5%)
Fibromyalgia	-	4 (4.1%)	-	14 (8.5%)	18 (3.2%)
Heart disease	-	-	-	35 (21.47%)	35 (6%)
Lack of comorbidities	13 (12.3%)	18 (18.4%)	43 (20.6%)	71 (43%)	145 (25.17%)

**Table 4:** Summary of studies assessing quality of life among people with lipoedema.

Reference	Year	Number of Subjects	The Aim of the Study	Quality of Life Questionnaire	Results
[27]	2018	*n* = 329	To investigate the relationship between depression and lowered self-esteem and the quality of life in people with lipoedema.	WHOQOL-BREF	Lower level of quality of life was associated with higher severity of symptoms, lower mobility, higher severity of depression, and lower self-esteem.
[26]	2021	*n* = 98	Examination of factors related to the quality of life in people with lipoedema, presentation of the sociodemographic characteristics, and clinical symptoms among Polish women with lipoedema.	WHOQOL-BREF	A low level of quality of life was observed in the subjects. With more and more symptoms, the quality of life is lower.
[23]	2018	*n* = 163	To investigate the characteristics of patients with lipoedema, quality of life, symptoms, and comorbidities.	RAND-36, EQ-5 D-3 L	The quality of life of people with lipoedema was significantly lower than the average quality of life in the Dutch population; people with comorbidities had a lower quality of life.

**Table 5:** Average time between first symptoms and diagnosis of lipoedema.

Reference	Average Age of Onset	Average Age of Diagnosis	Average Waiting Time for Diagnosis
[23]	20 years of age	38.3 years of age	18.3 years
[24]	16 years of age	31 years of age	15 years

## Data Availability

The data presented in this study are openly available in PubMed, Directory of Open Access Journals and Science Direct databases.
